# The Final Push for Polio Eradication: Addressing the Challenge of Violence in Afghanistan, Pakistan, and Nigeria

**DOI:** 10.1371/journal.pmed.1001529

**Published:** 2013-10-08

**Authors:** Seye Abimbola, Asmat Ullah Malik, Ghulam Farooq Mansoor

**Affiliations:** 1 National Primary Health Care Development Agency, Abuja, Nigeria; 2 Integrated Health Services, Islamabad, Pakistan; 3 Health Protection and Research Organization, Kabul, Afghanistan

## Abstract

Seye Abimbola and colleagues provide a view from Nigeria, Pakistan, and Afghanistan on global efforts to eradicate polio in those countries.

*Please see later in the article for the Editors' Summary*

**Summary Points**

- Polio eradication in Nigeria, Pakistan, and Afghanistan (the three remaining endemic countries) depends on understanding the common determinants and country-specific factors that underlie the failure to eradicate polio in these countries.
- Our review of the current situation suggests that the global health community and the governments of Afghanistan, Pakistan, and Nigeria need to build trust and to prioritise polio eradication as part of routine health services rather than highlighting it as “the only” health problem.
- Coercive strategies for making people take the polio vaccine and censorship of discussions around the controversies about polio vaccines need to be avoided.
- Because polio workers are a newly recognised soft target for anti-West terrorist groups in these countries, the publicity surrounding vaccination activities should be minimised.
- The global health community and national governments need to work directly with community members and their immediate leaders to dispel myths about polio vaccination rather than engaging only with regional or provincial religious leaders.

## Introduction

Over the last two years, the Global Polio Eradication Initiative (GPEI), a private-public partnership that has reduced polio worldwide by 99% since its launch in 1988, has greatly expanded the coverage of polio vaccination in Pakistan, Afghanistan, and Nigeria—the three countries where polio is still endemic and the success of the GPEI had previously been more limited. In Pakistan, the proportion of the highest-risk districts achieving the target vaccination threshold of 95% increased from 59% in January 2012 to a peak of 74% in October 2012 [1]. In Afghanistan, by the end of 2012, only about 15,000 children remained unreachable by vaccination workers, down from 80,000 in 2011; and in Nigeria, the proportion of high-risk local government areas where vaccine coverage reached the target threshold increased from 10% in February 2012 to 70% in February 2013 [1].

Recent fatal attacks on polio vaccination workers in politically fragile parts of Pakistan and Nigeria pose a serious threat to these gains and to the global eradication of polio—although so far the majority of children in these countries and Afghanistan are still being immunised, and polio campaigns continue. As researchers working in the polio endemic countries, which all have large Muslim populations, we acknowledge that increasing militancy, political unrest, lack of trust, and deteriorating security conditions are common denominators that threaten polio eradication efforts in all three countries. However, we also believe that the root causes of the failure of polio eradication differ markedly among these countries and are deeply embedded in country-specific contexts. It follows that the battle to eradicate polio will only be won with policies that are informed by a detailed understanding of these contexts. In this Policy Forum, we explore these differences and make policy proposals on how to respond to attacks on vaccination workers and to other factors that are impeding the final push for polio eradication.

## Afghanistan: Taliban Support for Polio Eradication

For several years, real and imagined wars have been fought with the West in politically fragile parts of Afghanistan, Pakistan, and Nigeria without significant and coordinated violence being aimed at vaccination workers. Indeed, when the Islamic fundamentalist Taliban regime in Afghanistan was in power from 1995 to 2001 it fully supported the GPEI, and likewise other warring groups active in Afghanistan—such as the global militant Islamist organisation Al Qaeda—were not interested in disrupting national polio eradication efforts. The recent attacks against polio workers in Pakistan and Nigeria may not be repeated in Afghanistan because of the re-emergence of the Taliban in this country and its ambition to regain its role in Afghan national politics. The Taliban is now working to build trust among the general population and have allowed local people to engage in social welfare campaigns, including the GPEI [2]. Importantly, whereas the Taliban in Pakistan is a militant group that is dependent upon a show of power to maintain their control over a small geographical area, the Taliban in Afghanistan is potentially the government-in-waiting.

In Kapisa province, northeast of Kabul, local Taliban members visit health facilities in the areas they control; they check the attendance of health workers to ensure they are always present to attend to patients (personal communication to GFM from Dr. Azizrurahman Safi, resident of Tagab district in Kapisa province, Afghanistan). They also play a key role in dispute resolution in rural areas where people feel that traditional laws are more responsive to their justice needs than modern laws [3]. Thus, GPEI program managers have been able to implement polio immunisation in politically unstable places through local people who also relate to the Taliban. This situation is fragile, however, because Taliban-like or pro–Al Qaeda forces entering Afghanistan from other parts of the world, particularly central Asian states, might hinder the GPEI by using interference with polio eradication as part of their strategy for countering the Western alliance in Afghanistan.

## Nigeria: The Dangers of Vertical Programming

The Islamist Boko Haram movement of northern Nigeria is not as organised as the Taliban or Al Qaeda, and there is little clarity about what they want from the Nigerian government [4],[5]. Boko Haram became prominent in 2011 amidst post-election violence, political disaffection, and distrust of the national government that followed northern Nigeria's unanticipated loss of hold on central presidential power [6]. This distrust of the government has been further fuelled by the GPEI. The people of northern Nigeria are beset by many problems, including poverty (up to three-quarters of people in several states in northern Nigeria live on less than one dollar a day) [7]; lack of sanitation, housing, and water supply; the three childhood killers (malaria, pneumonia and diarrhoea); malnutrition; and other health and developmental issues linked to poverty. There are many health issues in northern Nigeria that the people rightly consider as priority issues, but the government—which they do not trust—seems to concentrate on polio eradication. This situation breeds suspicions and leads people to refuse the polio vaccine to prevent a disease that is less threatening and less visible than other problems.

In this atmosphere of mistrust, militants and their conspirators who are eager to attract attention both within and outside of Nigeria can easily generate an offensive against health workers involved in polio eradication. They can convince a community that the health workers are agents of its enemy (the national government) who are working with another enemy, the West. The literal meaning of Boko Haram is “book is forbidden”; books for Boko Haram members are the pre-eminent symbol of Western education and, by extension, Western civilisation. Propaganda that the West is keen to reduce the population of Muslim communities through covert sterilisation campaigns disguised as immunisation outreaches also helps Boko Haram stir up feelings against polio vaccination workers. In the field experience of one of us (SA) it appears well known that some opinion leaders in northern Nigeria draw an association between Bill and Melinda Gates' commitment to polio eradication and contraception. They also seem to misunderstand the counterintuitive idea that, because couples tend to have fewer children when they realise their children are more likely to survive, successful vaccination programs in poor countries tend to reduce instead of increase their population.

## Pakistan: Nuisance Value and War Tactics

In Pakistan, polio workers had been performing the same role for the past 15 years without any substantial interference. Until recently, national immunisation days were considered a routine health-sector activity. However, in the past year polio workers have been attacked in the northwestern tribal areas and other parts of Pakistan. Just as in Nigeria, the attention paid to polio eradication in the international media and vertical programming may have led terrorist groups to believe that they can achieve some of their aims by interfering with polio eradication. Instead of fighting the military might of the USA and the Allied Forces, these groups may believe they can exploit the West's interest in eradicating polio. It is not known precisely who is behind the attacks on polio workers or why they have targeted this specific group of healthcare workers. However, the actions against polio workers may be driven by two objectives: first, to terrorise local populations and government workers, and second, to stop the house-to-house movement of polio workers who some terrorist groups suspect of carrying out surveillance activity to identify wanted persons (as was the case when the USA used a fake hepatitis B vaccination campaign to hunt Osama bin Laden) [8].

The local impact of attacks on polio workers has a shock value, an effective war tactic internationally [9]. As in northern Nigeria, where three North Korean doctors who had lived in the area since 2005 as part of a medical programme between the Yobe state government and the North Korean government were killed within a week of the attacks on polio workers [10], health workers in Pakistan have been targeted when they are involved in a polio campaign or are part of an international programme. Targeting the polio campaign is one way that terrorist groups can attract attention, not least because of the large-scale advertisement campaigns run by government authorities to sensitise local communities to polio vaccination.

## From Old Solutions to New Strategies for Polio Eradication

### Make Polio Eradication Part of Routine Immunisation

In our view, the ambition of the global health community to eradicate polio appears to be blinding it to lessons learned about health systems over the past 30 years. Polio eradication will only be achieved with stronger health systems and bottom-up community engagement, which is likely to require more time and more investment than is currently available in Pakistan, Nigeria, and Afghanistan because of their political fragility. The routine immunisation program is weak in Pakistan and Nigeria, in part because during polio immunisation campaigns many other programs stall [11]. The solution is to strengthen the routine health system, including door-to-door general vaccination coverage, rather than highlighting polio as “the only” health problem—an important solution that has been acknowledged in the Polio Eradication and Endgame Strategic Plan 2013–18 [1]. However, the time lag needed to put this solution into practice may be problematic. Although new opportunities often arise to integrate polio eradication activities into other immunisation campaigns, policy implementers frequently fail to take advantage of such opportunities. For example, during the recent measles outbreaks in Pakistan [12], polio vaccine could have been administered to millions of children in the affected districts but, as a routine practice, immunization campaigns were limited to vaccination against measles.

### Ensure That Locals See Polio Eradication as a Social Problem and Take Ownership

Current news reports in Pakistan suggest that the health workers who administer polio vaccines are or will be protected by security personnel. This is not a good image to portray in regions that have been torn apart by internal security threats, and where security has become a “personal” rather than a governmental responsibility. Moreover, until polio eradication is seen by the people as a social problem that deserves priority, it will continue to be part of a foreign agenda and the health workers involved in polio eradication will remain a low-paid cadre in the public health sector rather than agents of change. If local people can be encouraged to see polio eradication as a social problem, then groups such as Boko Haram and the Taliban will have an incentive to help secure access to vaccination as a means of winning people's support. We suggest that communication and implementation strategies that place polio eradication side-by-side with other health and development challenges faced by local communities should be introduced, an approach that the Polio Eradication and Endgame Strategic Plan 2013–18 also proposes [1]. In addition, local media should be encouraged to take ownership of polio eradication and highlight what has been achieved under the GPEI in each country, instead of concentrating on the negative consequences.

### Continue Immunisation But Without All the Fanfare

Some news stories have suggested that the government of Pakistan is making immunisation a condition for obtaining national identity cards and passports, as families with an unvaccinated child are denied these government documents [13]. Similarly, in Nigeria, there have been reports that the government plans to arrest and prosecute radio journalists who discussed controversies about polio vaccines on air [14]. Finally, there have been reports that the governments of Pakistan and Nigeria have promised security protection for polio workers in response to the recent killings [15], although the Nigerian government has decided against the move, which it argues will unduly militarise the programme [16]. The prime objective behind the attacks on polio campaigns is to create an environment of fear and anarchy, and it is essentially impossible to provide security protection for hundreds of thousands of health workers. Therefore, one way to facilitate polio eradication might be to continue with vaccination activities but with less publicity, and without the involvement of coercion or military strategies. Because it will be difficult for government agencies to maintain neutrality and keep a low profile if security is provided for health workers via military personnel, we believe that efforts should be made to depoliticise polio activities. Support for this approach comes from rural areas in Afghanistan, which have been the most insecure places for government employees to work but where negotiations with and empowerment of local communities has facilitated the national polio eradication initiative [2].

### Work Directly with Community Members and Leaders

Polio vaccination was initially rejected in northern Nigeria in 2003–04 following rumours that the vaccine contained chemicals that would sterilise female children and reports that an unregistered drug used in northern Nigeria during a meningitis outbreak in 1996 left several children paralysed [17],[18]. The strategy that helped resolve the initial rejection of the polio vaccine was to work with traditional religious leaders in northern Nigeria to improve the acceptance of the vaccine. However, we believe that this strategy can only go so far. Although people will initially listen to their leaders, as the leaders receive more funds people will increasingly see them as agents of the government, which they do not trust. This problem might be mitigated by focusing on community members and leaders of smaller community units, such as village health committees, in addition to engaging provincial, traditional, and religious leaders. Support from Saudi Arabia and its health authorities cannot be underestimated in being able to reach out to Muslim leaders internationally, thereby giving the final push for polio eradication an Islamic face [19]. In addition, there is a need to use community networks to monitor and vaccinate along the porous border between Afghanistan and Pakistan as cross-border movements lead to re-infections between the two countries.

### Build Trust by Keeping a Low Profile on International Deadlines

It is good news that the GPEI has recently announced that the new target for polio eradication is 2018 [20]. However, 2018 may be too soon given the weak health systems and other characteristics of Nigeria, Pakistan, and Afghanistan. The withdrawal of international forces in 2014 from Afghanistan may also complicate the security situation in Afghanistan and hinder its relatively successful efforts in the fight against polio [2]. It might be better if there were no deadlines at all, but we appreciate that a clear deadline can concentrate global polio eradication efforts. However, we are also aware that international goals and the national or local ones may conflict. We suggest, therefore, that the GPEI and its major players, such as the Bill & Melinda Gates Foundation, Rotary International, UNICEF, WHO, and the US Centers for Disease Control and Prevention, keep a low profile on the deadlines and instead focus their energies on supporting national and sub-national governments to strengthen routine immunisation and other primary health care services. We believe that giving the GPEI a lower international profile could weaken the “Western” link, and help to build trust both in polio immunisation and in national governments. Trust is essential if communities are to accept the vaccines and is needed to ensure the commitment of local leaders to polio eradication. Trust on its own will not stop the attacks on polio workers, but once terrorist groups realise that polio eradication no longer has a high profile, they may turn their attention to other soft targets. Ultimately, all these soft targets must be protected by ensuring that, as with the Taliban in Afghanistan, local aggrieved groups see the protection of people's access to social welfare services such as immunisation as a tool rather than a hindrance to acquiring legitimacy.

## Conclusion

The aims of our proposals are the same as those identified by the GPEI in its Polio Eradication and Endgame Strategic Plan 2013–18 [1], but the specific strategies and tactics we propose are tailored to the realities that we experience as health researchers and practitioners in our countries. Misinformation and myths around immunisation are neither peculiar to the polio vaccine nor to Nigeria, Pakistan, and Afghanistan, but we believe that implementation of the GPEI has been greatly limited in these countries because of a lack of trust between the people and their national, West-supported governments. It takes time to build trust. Already, the support of local people has enabled children in insecure areas of these countries to be reached; but an increase in the coverage of overall health services and improved access to health services by the general population might help in building trust between governments and the people and between health workers and communities. Without the nuanced and realistic approach to policy-making and global goal-setting that we have advocated in this article, we are concerned the recent portrayal of polio by some as the new battleground between Western forces and terrorist groups may change the perception and resolution of high-risk public health problems in the same way that suicide bombings have changed war tactics and security requirements.

## Abbreviations

GPEI: Global Polio Eradication Initiative
